# High glucose increases LPS-induced DC apoptosis through modulation of ERK1/2, AKT and Bax/Bcl-2

**DOI:** 10.1186/1471-230X-14-98

**Published:** 2014-05-28

**Authors:** Mei Feng, Juan Li, Jun Wang, Chunyan Ma, Yulian Jiao, Yan Wang, Jie Zhang, Qiuying Sun, Ying Ju, Ling Gao, Yueran Zhao

**Affiliations:** 1 Central Laboratory, Shandong Provincial Hospital affiliated to Shandong University, Jinan, China; 2 Department of Obstetrics and Gynecology, Shandong Provincial Hospital affiliated with Shandong University, Jinan, China; 3 Jinan Central Hospital, Affiliated with Shandong University, Jinan, China; 4 Department of Occupational Environmental Health Monitoring and Evaluation, Shandong Center for Disease Control and Prevention, Jinan, China; 5 Department of Endocrinology, Shandong Provincial Hospital affiliated with Shandong University, Jinan, China; 6 Department of Clinical Laboratory, Shandong Provincial Hospital affiliated with Shandong University, Jinan, China

**Keywords:** Dendritic cell, Apoptosis, Diabetes mellitus, Glucose, Gastrointestinal complications diabetes

## Abstract

**Background:**

This study investigates the effect of glucose on the LPS-induced apoptosis of dendritic cells in the intestinal tract of mice and the dendritic cell line DC2.4.

**Methods:**

Flow cytometry was used to detect dendritic cell apoptosis both *in vivo* and *in vitro*. Hoechst 33258 staining was used to detect the morphological changes characteristic of apoptotic nuclei. Expression of apoptosis related proteins was investigated by western blot analysis and immunohistochemistry.

**Results:**

Pretreatment with a high concentration of glucose increased apoptosis of LPS-treated dendritic cells both *in vivo* and *in vitro* at 24 h. No effect was evident at the earlier time points of 15 min and 6 h *in vitro*. Furthermore, at 24 hours the expression of the survival proteins AKT, ERK and Bcl-2 was decreased, while the expression of the proapoptotic protein Bax was increased. AKT, ERK, Bcl-2 and Bax were mainly located in the cytoplasm by immunohistochemistry.

**Conclusions:**

These results suggest that high glucose concentrations might prime dendritic cells for apoptosis induced by LPS in the intestinal tract through upregulating the expression of Bax and downregulating the expression of AKT, ERK and Bcl-2. Therefore, this study may give clues to understanding the immunological mechanism behind gastrointestinal complications in diabetes mellitus.

## Background

There were approximately 285 million adults with diabetes worldwide in 2010, and the number has continued to increase [1]. Diabetes can lead to premature death and has various complications associated with it including blindness, peripheral neuropathy, renal disease and cardiovascular diseases. The complications of diabetes mellitus are accelerated in people who also have poorly controlled blood sugar levels. Other risk factors for complications include smoking, elevated cholesterol levels, obesity, high blood pressure and lack of regular exercise [2]. It has also been reported that high glucose levels induce immune cell apoptosis [3]. Raab et al. additionally showed that high energy substrates in the diet increased apoptosis in the intestinal epithelium [4].

The immune system is pivotal in mediating interactions between the host and the microbiota that shape the intestinal environment [5,6]. Furthermore, dendritic cells (DCs) are pivotal in tolerance induction and direct the differentiation of T cells in the intestine. Therefore, DC apoptosis will result in immunosuppression, promote regulatory T cell (Treg) generation and induce functional impairment of remaining DCs [7,8]. Additionally, the gastrointestinal tract is continuously exposed to foreign antigens including invading bacteria and viruses, food antigens and also potentially altered-self antigens such as tumor antigens. Infection with certain bacteria and other pathogens can induce DC apoptosis, which will result in the dysregulation of the immune balance in the gastrointestinal tract [9]. Thus, deeper understanding of the mechanism regulating intestinal DC apoptosis is needed.

It has been shown that PI3K (phosphatidylinositol-3-kinase)/AKT and MEK (MAP/ERK kinase)/ERK signaling is involved in the regulation of bone marrow-derived DC (BMDC) apoptosis, as inhibiting both PI3K and MEK resulted in 70% of the DCs undergoing apoptosis [10]. Furthermore, AKT (also known as protein kinase B, PKB) and ERK (Extracellular signal-regulated protein kinases) are known regulators of DC cell survival [11]. Additionally, it is also known Bax (Bcl-2 Associated X Protein) and Bcl-2 (B cell lymphoma/lewkmia-2) play a central role in regulating apoptosis [12]. Increased expression of Bcl-2 and decreased expression of Bax promotes cell survival by inhibiting apoptosis [13].

As far as we know, there are no reports on the effect of high glucose concentrations on DC apoptosis in the mouse intestine. We hypothesized that high concentrations of glucose might accelerate LPS (Lipopolysaccharides) induced DC apoptosis in the mouse intestine through alteration of the activation of ERK, AKT, Bax and Bcl-2. We found that high glucose did indeed increase LPS-induced DC apoptosis. This effect was mediated through inhibition of AKT and ERK phosphorylation, increased Bax expression and decreased Bcl-2 expression. This study may give clues to understanding the mechanism behind the immunological changes present in the gut in diabetes mellitus.

## Methods

### Experimental animals

Male BALB/c mice, 10 weeks old, were purchased from the Animal Center at Shandong University. All experimental procedures were approved by and performed according to the guidelines of the animal ethics committee of the Shandong University School of Medicine. In brief, mice were injected intraperitoneally with streptozotocin (STZ, 60 μg/g) once a day for 5 days to induce diabetes [14,15]. Control mice were injected with PBS (Phosphate buffer saline). Seven days after the final injection, blood glucose levels were examined by glucometer (UltraVue, Johnson & Johnson, New Brunswick, NJ, USA). Mice with blood glucose levels greater than 300 mg/dl were considered diabetic. Diabetic mice were given LPS (1 mg in 500 μL PBS) orally once to induce inflammation on the 19th day after the first streptozotocin injection, while control mice were treated with the same volume of PBS. 24 hours later mice were killed and intestines dissected and digested with collagenase. Immune cells were isolated by layering on a Percoll gradient, then DCs were further purified using CD11c magnetic beads (130-052-001, Miltenyi Biotec Inc, Bergisch Gladbach, Germany) [16,17]. Finally the DCs were collected, stained and analyzed by flow cytometry.

### Cell culture

Dendritic cells (DC2.4 cell line) were cultured on collagen coated dishes in complete medium containing RPMI-1640, 10% fetal bovine serum, 1 × 10^4^ units/ml penicillin and 10 mg/ml streptomycin. Cells were passaged using standard cell culture techniques. Culture dishes were placed in an incubator equilibrated with 5% CO_2_ at 37°C. The medium was refreshed at intervals of 3 days. To maintain uniform condition, the cells in passages 20 to 30 were used for experiments.

### Cell treatment

DC2.4 cells were treated with a high dose of glucose (25 mmol/L) for 48 h. Then LPS (100 μg/ml) was added to detect whether the glucose concentration could affect DC apoptosis. After 24 h LPS treatment (cells grown to approximately 80% confluence), the cells were harvested for both western blot analysis and flow cytometry.

### Analysis of apoptosis by flow cytometry

Apoptosis of dendritic cells was determined using an Annexin-V and PI apoptosis kit (BU-ap0102, Life Technologies, Carlsbad, CA, USA). After washing twice with PBS, cells were re-suspended in binding buffer and incubated with Annexin-V and PI for 15 min. At least 5000 cells were counted per sample. Apoptotic cells appeared as Annexin-V positive and PI negative.

### Analysis of apoptosis by Hoechst 33258 staining

Hoechst 33258 staining (B33258, Sigma, St Loius, MO, USA) was used to visualize the morphological changes in apoptotic cell nuclei. After being washed with PBS and fixed with 4% paraformaldehyde for 30 min, 0.5 μg/ml Hoechst 33258 was added to the wells and incubated for 5 min. Then cell nuclei were analyzed by fluorescence microscopy. Apoptotic cells were characterized by chromatin condensation and multiple chromatin fragments.

### Western blot

Cells were collected and washed three times with PBS before lysis in lysis buffer (MK163780, ThermoFisher Scientific Inc, Waltham, MA, USA). The protein concentration of the lysates was determined according to the BCA method using the Protein Quantitative Analysis kit (2812 k; CWBIO, CoWin Biotech Co. Ltd., Beijing, China). For electrophoresis, 100 μg of protein was loaded into the wells of a 10% SDS polyacrylamide gel. After separation, proteins were transferred to nitrocellulose membranes, blocked for 3 h at room temperature in blocking buffer (5% nonfat dry milk, Tween-Tris-buffered saline), washed in PBS 3 times and incubated with primary antibody of ABCAM (Rabbit polyclonal to ERK, ab16869, 1: 1:5000; Rabbit polyclonal to AKT, ab66138, 0.15 μg/ml; Rabbit polyclonal to Bax, ab7977, 1:1000; Rabbit polyclonal to Bcl-2, ab18210, 1 μg/ml) and Actin (sc-1616, 1:200, SANTA CRUZ BIOTECHNOLOGY) overnight at 4°C with gentle agitation. Next day after washing three times in PBS, the nitrocellulose membranes were blotted for 1 h at room temperature with secondary antibodies (ab136817, 1:5000, abcam). Finally, immunolabeled protein bands were detected using the ECL(Electro-Chemi-Luminescence) method (Immobilon™ Western, Millipore Corporation, Billerica, MA, USA) and quantified using an Alpha Imager 2200 (ProteinSimple, Santa Clara, CA, USA).

### Immunohistochemistry

DC2.4 cells were treated as described above. After fixing in 4% paraformaldehyde for 15 min and washing with PBS, cells were permeabilized with 0.1% triton X-100 for 15 min. 3% hydrogen peroxide was used to block endogenous peroxidases. After washing in PBS and blocking in 10% goat serum, cells were incubated with primary antibody of ABCAM (Rabbit polyclonal to ERK, ab16869, 1:1000; Rabbit polyclonal to AKT, ab66138, 1 μg/ml; Rabbit polyclonal to Bax, ab7977, 1 μg/ml; Rabbit polyclonal to Bcl-2, ab18210, 5 μg/ml) and Actin (sc-1616, 1:250, SANTA CRUZ BIOTECHNOLOGY) overnight in a humidified chamber at 4°C. The next day, the cells were washed before being incubated with secondary antibody (SP rabbit HRP kit, CW2035, CWBIO) for 30 min at room temperature. After washing, positive immunostaining was revealed by the DAB(3,3’-diaminobenzidine) method (D8001, Sigma). PBS was used as a negative control for the primary antibodies and Actin was used as a positive control.

### Statistical analysis

Data were analyzed with SigmaStat 3.5 software (SYSTAT Software, San Jose, CA, USA). One-way ANOVA analysis was followed by Dunnett’s test to analyze the differences among the groups. Data are presented as the mean ± SE of at least four independent experiments. *P* < 0.05 was considered a statistically significant difference.

## Results

### Effects of glucose on LPS-treated dendritic cell apoptosis

**Figure 1:**
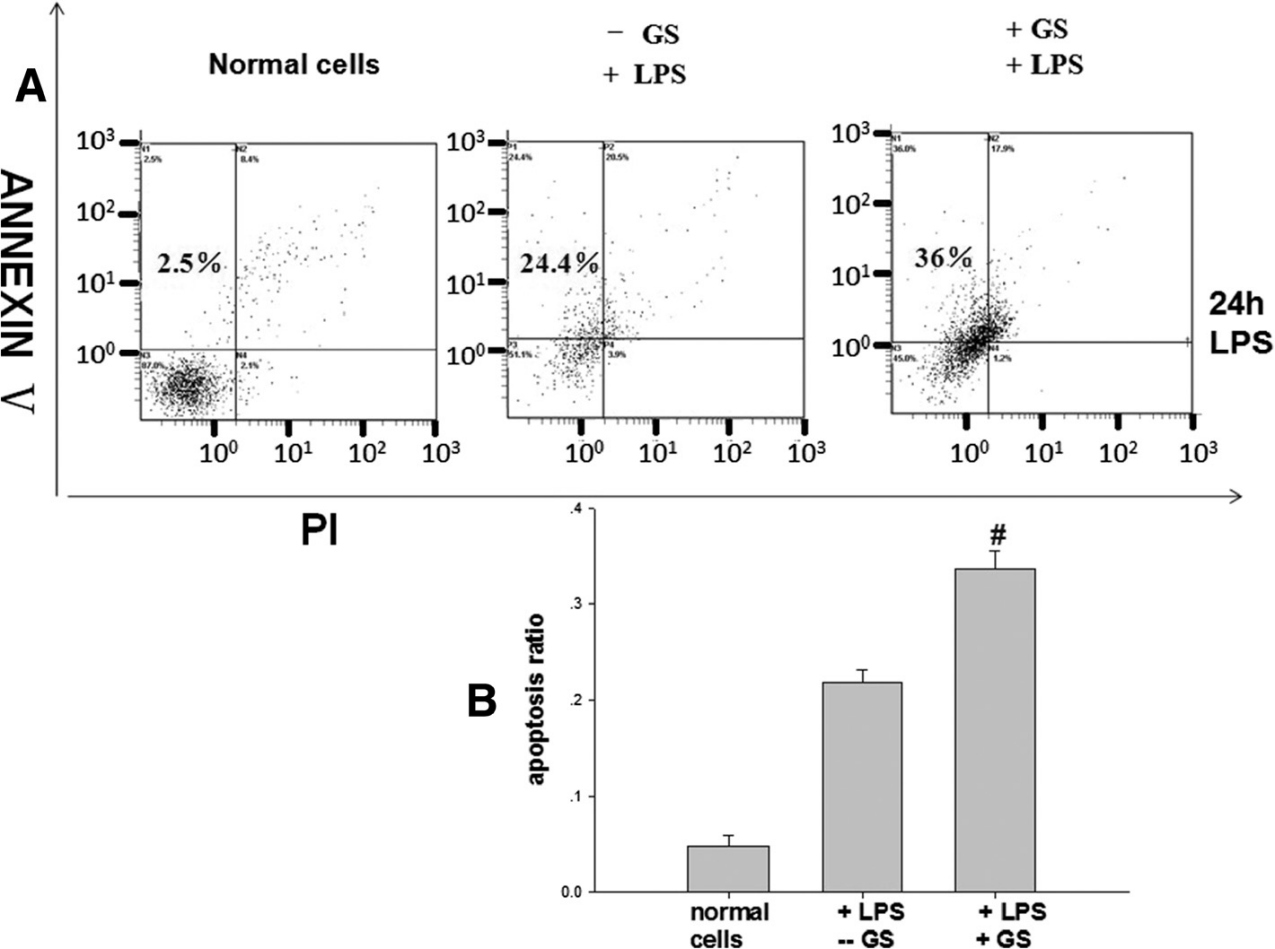
**The effects of high glucose on LPS-induced dendritic cell apoptosis in mouse intestinal tract *in vivo*. (A)** A representative FACS plot of intestinal DC apoptosis at 24 h. The upper left quadrant represents the apoptotic cells (Annexin-V^+^ (An^+^) PI^-^); the lower left is live cells (An^-^ PI^-^); the upper right is dead cells (An^+^ PI^+^). **(B)** Summary of intestinal DC apoptosis from mice administrated LPS 24 h previously. STZ treated mouse were used as the high glucose group. High glucose significantly increased apoptosis of intestinal DC, #*p* < 0.05 vs. control mice.

**Figure 2:**
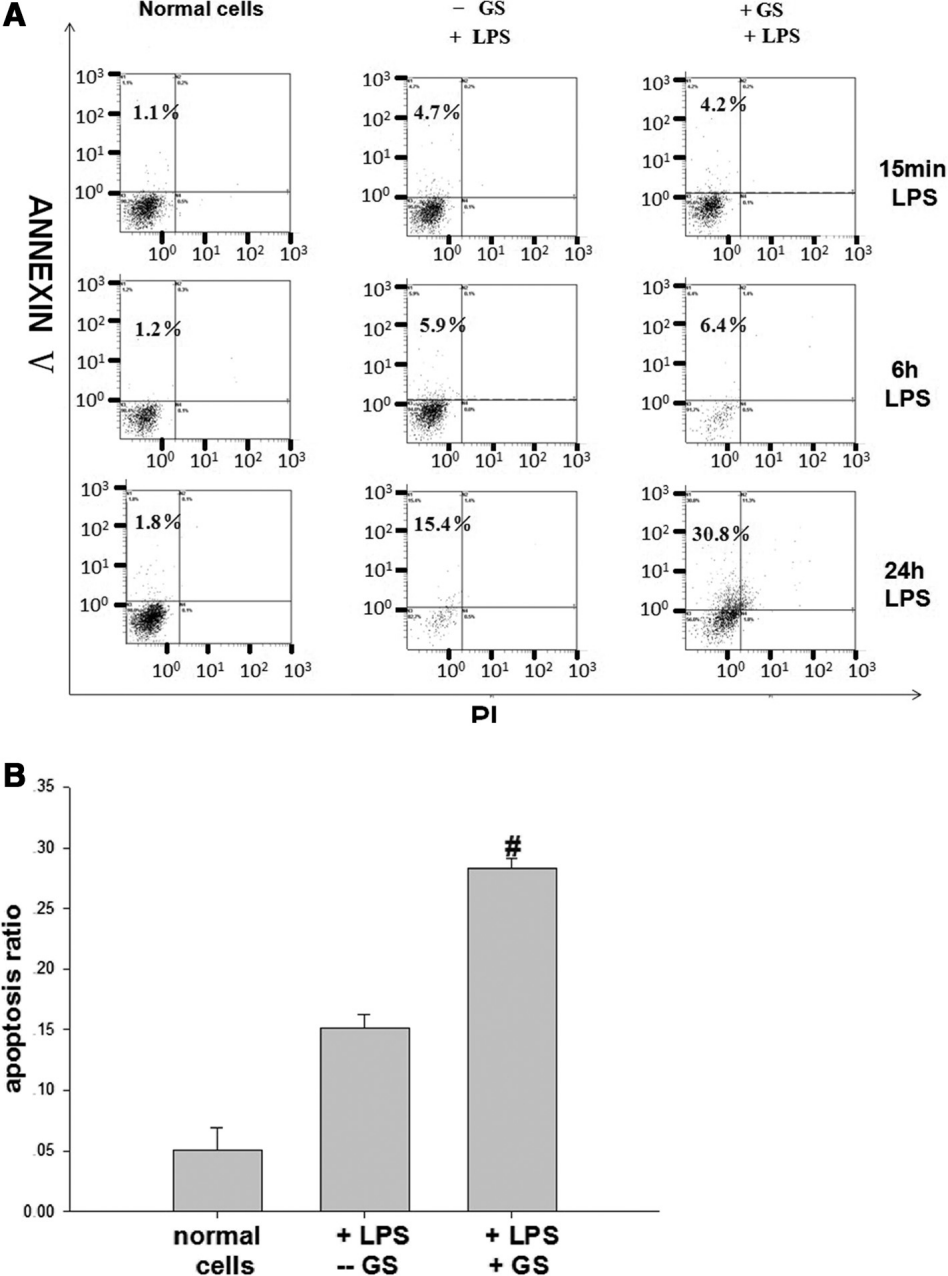
**The effects of glucose on LPS-induced apoptosis in DC2.4 cells *in vitro*. (A)** A representative figure of apoptosis induced by LPS treatment at 15 min, 6 h and 24 h. **(B)** Histogram of the LPS-induced apoptosis of DC2.4 cells at 24 h. Glucose pre-administration markedly elevated apoptosis of cells treated with LPS for 24 h, but this effect was not evident in cells treated for only 15 min or 6 h, #*p* < 0.05 vs. control mice.

**Figure 3:**
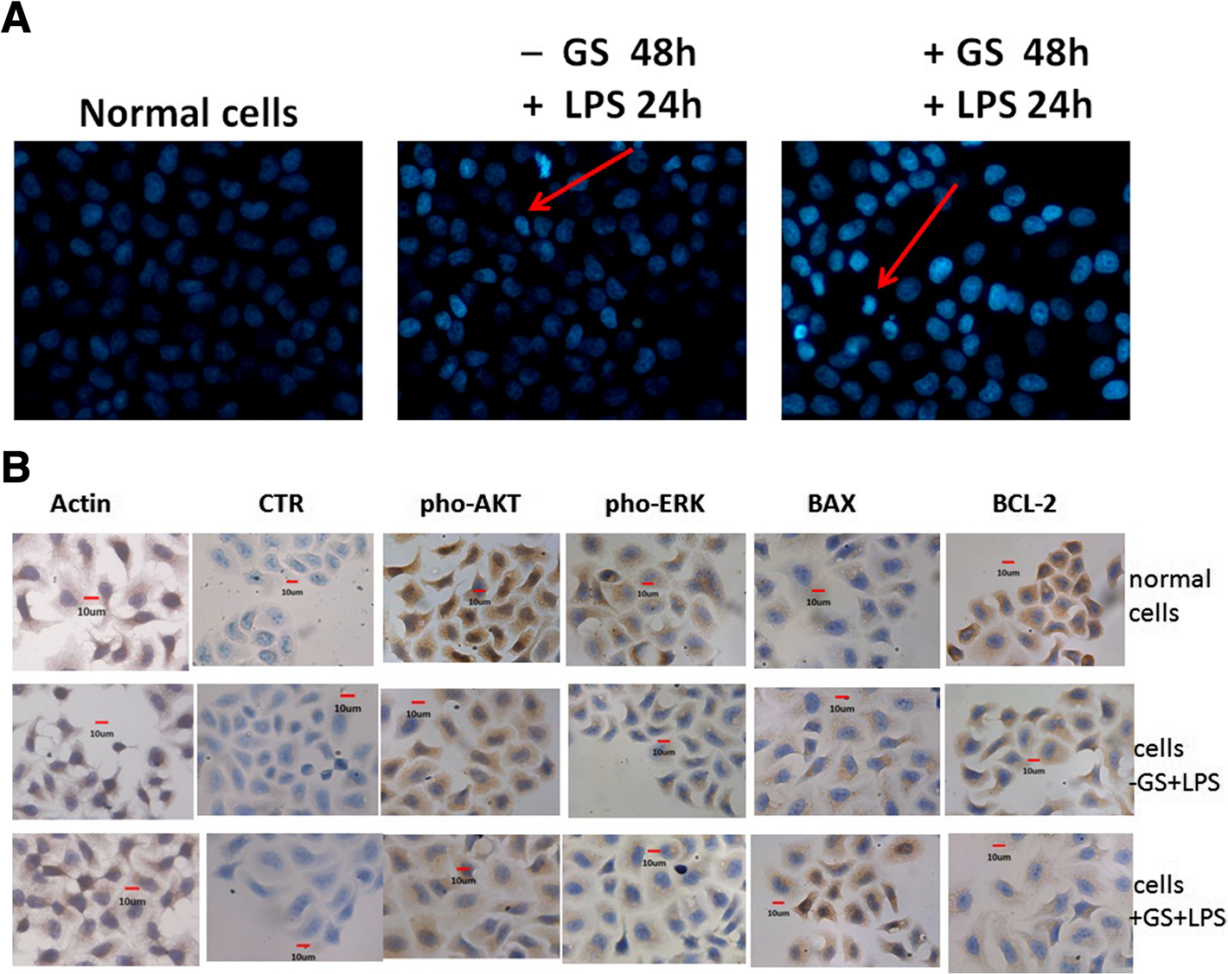
**The effects of glucose on the nuclear morphology of LPS-treated DC2.4 cells *in vitro*. (A)** Hoechst 33258 staining of DC2.4 cells treated with LPS for 24 h (400×). Cells treated with a high concentration of glucose showed obvious apoptotic features including cytoplasm concentration, chromatin condensation and multiple chromatin fragments compared with cells without glucose. Brightly stained nuclei represent cells undergoing apoptosis. **(B)** Localization of AKT, ERK, Bax and Bcl-2 in DCs (400×). CTR is negative control treated with the second antibody alone. No immunoreactive cells were detected in the CTR. Actin staining was used as positive control. High glucose elevated Bax expression but decreased the expression of Bcl-2 and the phosphorylation of AKT and ERK.

Untreated cells had a background level of less than 5% apoptosis both *in vivo* and *in vitro*. In cells treated with LPS for 24 h, glucose pre-administration markedly elevated the level of apoptosis (Figure 1 and Figure 2, *p* < 0.05) while glucose alone had no statistically significant effect (data not shown). However, increased apoptosis was not evident at 15 min and 6 h in LPS treated cells (Figure 2A).To determine the effects of glucose on apoptosis, cells were exposed to glucose for 48 h. Then cell nuclei were stained using Hoechst 33258 and analyzed by fluorescence microscopy. As shown in Figure 3A, cells treated with glucose showed obvious apoptotic features including cytoplasm concentration, chromatin condensation and multiple chromatin fragments. In normal control cells, nuclei were distributed uniformly and were larger in size compared to the other two treated groups. As for these two treated groups, the cells pre-treated with glucose appeared to have a higher degree of apoptosis.

### Apoptosis related protein expression in dendritic cells

**Figure 4:**
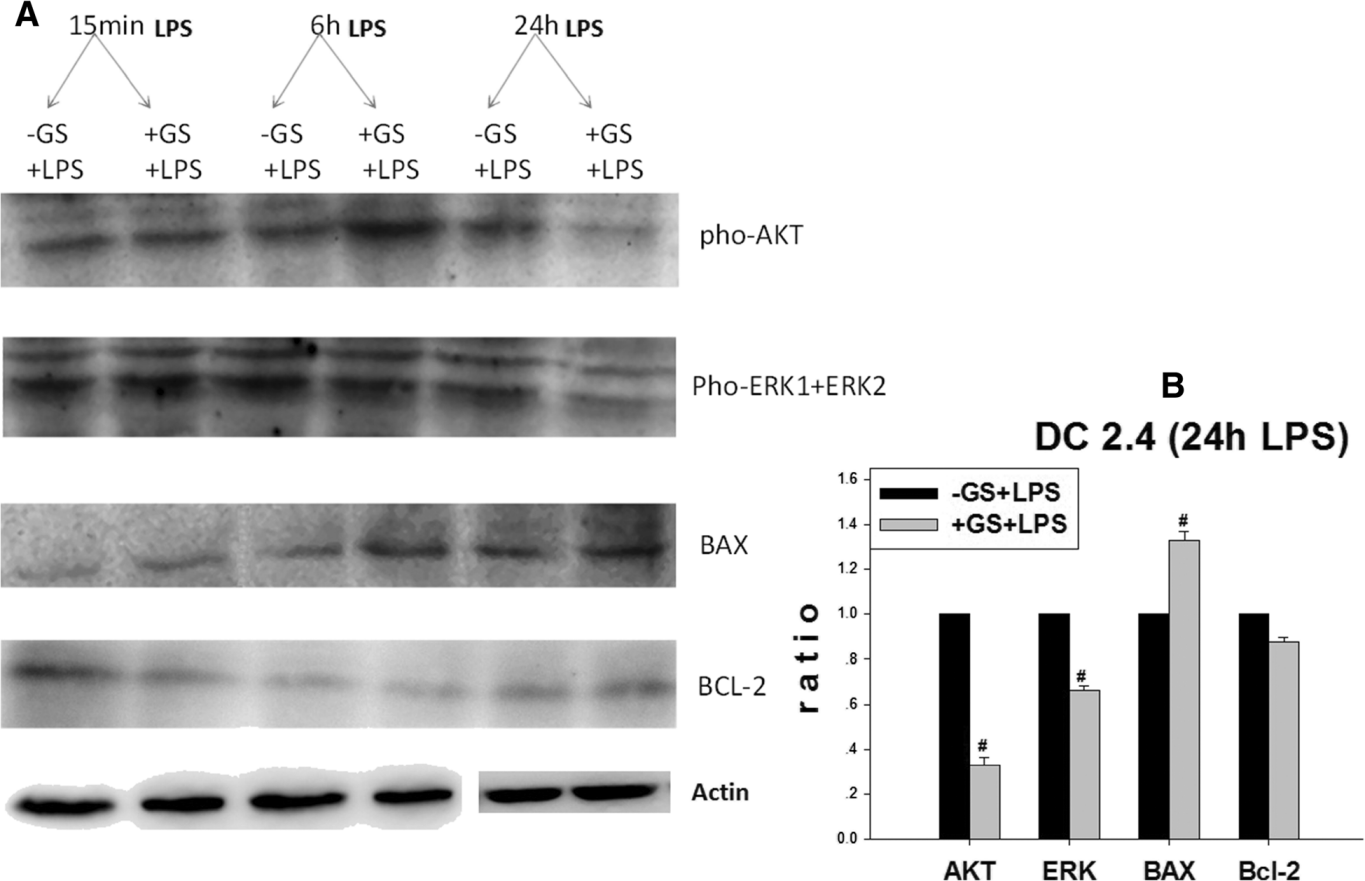
**Effects of glucose on the expression of apoptosis related factors *in vitro. *(A)** Effects of glucose on the protein expression of AKT, ERK, Bax and Bcl-2 in DCs treated with LPS for different periods of time. At high glucose concentrations at 24 h LPS stimulation, the protein expression of AKT, ERK and Bcl-2 decreased, while BAX increased. These effects were not evident in cells treated with LPS for 15 min and 6 h. Actin is internal control. Figure 5.**B** is the statistical chart. Gray values of target bands were divided by Actin to obtain normalized data. #*p* < 0.05 vs. control mice.

**Figure 5:**
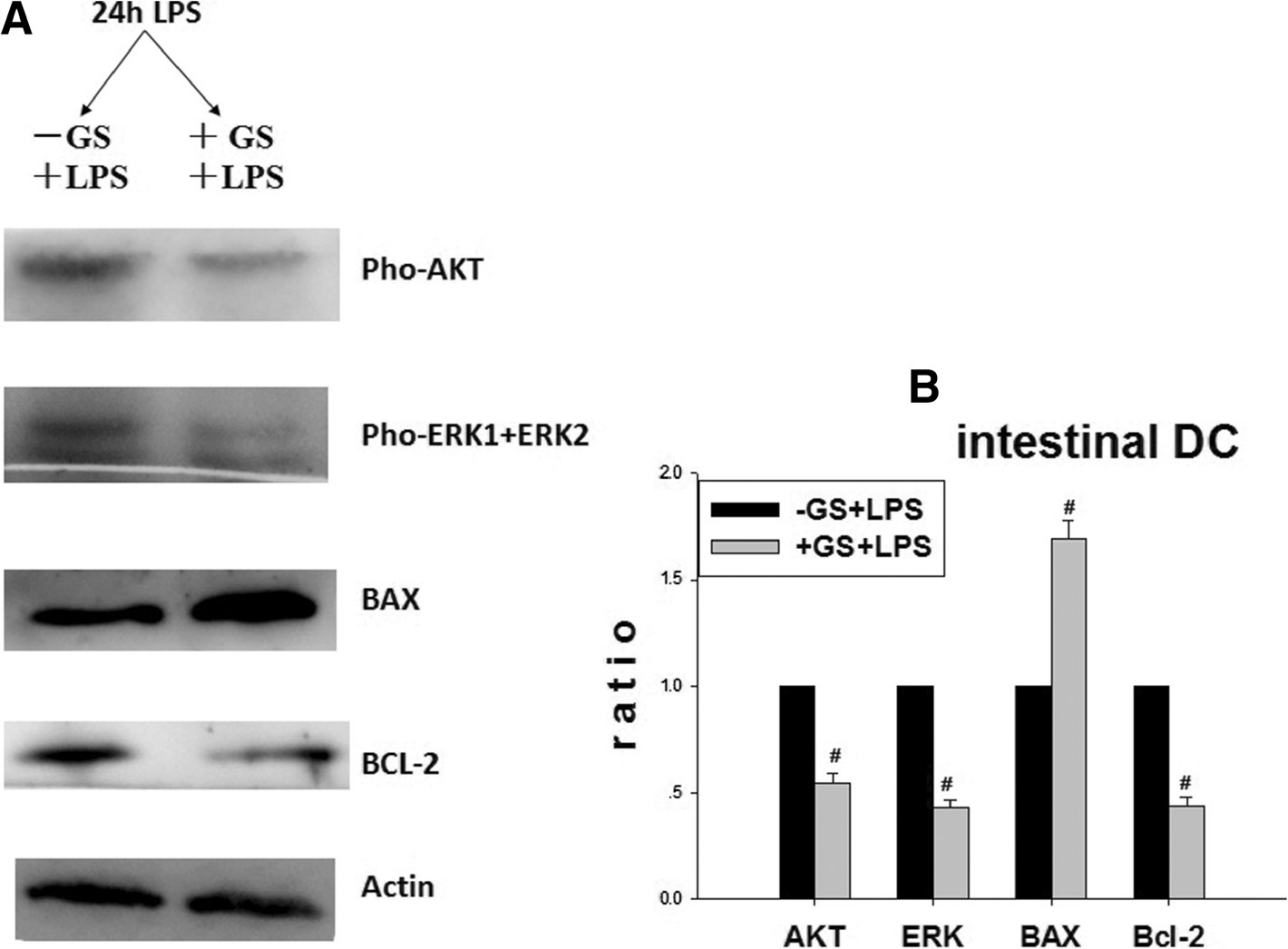
**Effects of glucose on the expression of apoptosis related factors *in vivo.*** Actin is internal control. High glucose could elevate intestinal DCs apoptosis of 24 h LPS treated diabetic mouse (Figure 5.**A**). Figure 5.**B** is the statistical chart. There was significant difference between the diabetic mouse and normal mouse. Gray values of target bands were divided by Actin to obtain normalized data. # *p* < 0.05 vs. control mice.

As shown in Figure 4, glucose pre-administration could markedly reduce the levels of phosphorylated (pho)-AKT, pho-ERK and Bcl-2 in cells treated with LPS for 24 h *in vitro*. However, these changes were not seen in the 15 min and 6 h treated groups. With regards to the expression of Bax, glucose could markedly elevate its expression in cells treated with LPS for 24 h. In addition, similar effects were found in diabetic mouse *in vivo* that AKT, pho-ERK and Bcl-2 were decreased and Bax was increased Figure 5. These results suggest that the increase in LPS-induced DC apoptosis in high glucose primed cells was mediated through AKT, ERK and Bax/Bcl-2 pathways.

### Location of apoptosis related proteins in dendritic cells

The immunoreactivity for the apoptosis related proteins (AKT, ERK, Bax, Bcl-2) were mainly located in the cytoplasm (Figure 3B). The negative control showed no background staining. Glucose administration decreased the number of positive cells for the antiapoptotic proteins AKT, ERK and Bcl-2 and also reduced the intensity of immunoreactivity of the labeled cells. In comparison, both the number and intensity of the Bax immunoreactive cells was increased.

## Discussion

The present study demonstrates that LPS-treated dendritic cells in the intestine of diabetic mice undergo increased apoptosis compared with normal mice. A similar effect was observed in the DC2.4 cell line *in vitro*. High glucose concentration increased the level of apoptosis of LPS-treated DCs via alterations in the AKT, ERK, Bax and Bcl-2 pathways. AKT, ERK Bax and Bcl-2 were mainly located in the cytoplasm of DCs.

LPS, the prototypical endotoxin, is the major component of the outer membrane of Gram-negative bacteria and through TLR4 can trigger both proapoptotic and antiapoptotic pathways [18]. LPS can induce PBMC-derived DC apoptosis [19,20], although other studies found no signs of apoptosis in LPS-treated DCs [21,22]. The Enterobacteriaceae are a large, heterogeneous group of gram-negative bacilli whose natural habitat is the intestinal tract of humans and animals. These bacteria will cause disease when the immune balance is disrupted. Therefore we used LPS to mimic the presence of bacteria in the intestine to explore if high glucose can affect DC survival in the inflamed intestine. We found that LPS could elevate intestinal DC apoptosis. Furthermore, LPS could also increase DC2.4 apoptosis in a dose-dependent fashion (data not shown).

AKT and ERK proteins are known regulators of cell survival. AKT, a serine/threonine-specific protein kinase, plays as a central role in the signaling pathways regulating metabolism and cellular transformation. AKT can regulate cell growth and apoptosis by activating a series of downstream signaling molecules [23]. It has been shown that DC maturation and antiapoptotic responses are associated with activation of AKT signaling. Blocking AKT signaling impairs DC maturation and induces apoptosis [24]. ERK is an important mediator of BMDC activation [25]. The Raf/MEK/ERK signaling pathway acts as an important element in DC generation and differentiation [10]. In the present study, we found that LPS decreased the phosphorylation of AKT and ERK1/2 in DCs at 24 h. If the cells were pretreated with high glucose, there was a greater decrease in the phosphorylation of these survival factors.

The Bax/Bcl-2 family of proteins highlights the complexity of cellular biology. Bcl-2, an anti-apoptotic protein, is known to be a negative regulator of apoptosis which can prevent cytochrome c release from mitochondria and protect DNA from fragmentation. In cell culture, transfecting Bcl-2 into U937 cells protected the cells from apoptosis induced by various insults [26]. Conversely Bax, a pro-apoptotic member of the Bax/Bcl-2 family, may be a key factor promoting cytochrome c release. The ratio of Bax to Bcl-2 is therefore a crucial determinant of apoptosis in the cell. In the present study, it was demonstrated that LPS increased the expression of Bax protein and decreased the expression of Bcl-2 protein in DCs at 24 h. If the cells were pretreated with high glucose, the ratio of Bax/Bcl-2 was further increased.

## Conclusions

Taken together, our results showed that high glucose levels present in diabetic mice could increase the LPS-induced apoptosis of DCs in the inflamed intestine. Apoptosis related proteins such as AKT, ERK, Bax and Bcl-2 regulated LPS-induced apoptosis in DCs. Specifically, high glucose concentrations increased LPS-induced apoptosis in DCs by enhancing the down-regulation of AKT and ERK phosphorylation and the up-regulation of the Bax/Bcl-2 ratio. These results contribute to a greater understanding of the mechanism behind the immunological changes in the gastrointestinal tract of patients with diabetes mellitus.

## Abbreviations

DC: Dendritic cell; LPS: Lipopolysaccharides; AKT: Serine/threonine protein kinase/known as Protein Kinase B; ERK: Extracellular signal-regulated protein kinases; Bax: Bcl-2 Associated X Protein; Bcl-2: B cell lymphoma/lewkmia-2.

## Competing interests

The authors declare that they have no competing interests.

## Authors’ contributions

MF conceived and designed the experiments, carried out the experimental work and drafted the manuscript. JL carried out the flow cytometry and statistical analysis. JW took care of and obtained samples from mice. CM carried out protein detection and statistical analysis. YJ participated in analysis and interpretation of results. YW participated in analysis and interpretation of results. JZ contributed reagents and materials. QS performed data collection and statistical analysis. YJ contributed materials and drafted the manuscript. LG participated in paper design and coordination and helped to draft the manuscript. YZ participated in paper design and coordination and helped to draft the manuscript. All authors read and approved the final manuscript.

## Pre-publication history

The pre-publication history for this paper can be accessed here:

http://www.biomedcentral.com/1471-230X/14/98/prepub
